# Bending impact on the performance of a flexible Li_4_Ti_5_O_12_-based all-solid-state thin-film battery

**DOI:** 10.1080/14686996.2018.1468199

**Published:** 2018-05-25

**Authors:** Alfonso Sepúlveda, Jan Speulmanns, Philippe M. Vereecken

**Affiliations:** a Imec, Leuven, Belgium; b Centre for Surface Chemistry and Catalysis, University of Leuven, Leuven, Belgium

**Keywords:** Flexible all-solid-state thin-film battery, thin-film LiPON, thin-film Li_4_Ti_5_O_12_, bending state, stress/strain induced, rate performance, 50 Energy Materials, 107 Glass and ceramic materials, 206 Energy conversion / transport / storage / recovery, 207 Fuel cells / Batteries / Super capacitors, 306 Thin film / Coatings

## Abstract

The growing demand of flexible electronic devices is increasing the requirements of their power sources. The effect of bending in thin-film batteries is still not well understood. Here, we successfully developed a high active area flexible all-solid-state battery as a model system that consists of thin-film layers of Li_4_Ti_5_O_12_, LiPON, and Lithium deposited on a novel flexible ceramic substrate. A systematic study on the bending state and performance of the battery is presented. The battery withstands bending radii of at least 14 mm achieving 70% of the theoretical capacity. Here, we reveal that convex bending has a positive effect on battery capacity showing an average increase of 5.5%, whereas concave bending decreases the capacity by 4% in contrast with recent studies. We show that the change in capacity upon bending may well be associated to the Li-ion diffusion kinetic change through the electrode when different external forces are applied. Finally, an encapsulation scheme is presented allowing sufficient bending of the device and operation for at least 500 cycles in air. The results are meant to improve the understanding of the phenomena present in thin-film batteries while undergoing bending rather than showing improvements in battery performance and lifetime.

## Graphical abstract

**Figure:**
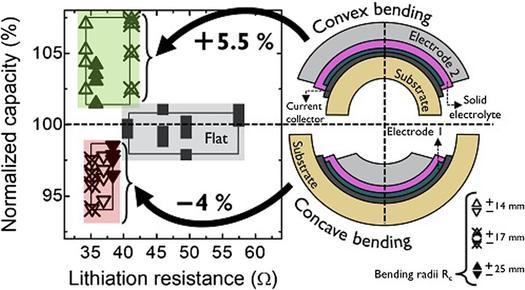

## 1. Introduction

Providing sufficient electrical energy storage is one of the key challenges for the next century. A shift from fossil to renewable energies, development of practical electrical vehicles, mobile electronics, and Internet of Things devices lead to a strong demand of high-energy, high-power, high-rate capability, long lifetime, high-output voltage, safe, low-cost, environment-friendly, and rechargeable batteries. Lithium (Li) and Li-ion batteries (LIBs) are outperforming most alternative battery chemistries. Li chemistry provides much higher power and energy densities in both gravimetric and volumetric terms which are the most important parameters for applications in portable electronics such as smart phones, digital cameras, and laptops [1]. Conventional batteries using liquid electrolyte present inherent risks like leakage, flammability, and formation of Li dendrites, which can lead to electrical shorts resulting in explosion of the battery. To overcome these issues solid-state electrolytes are introduced. Furthermore, all-solid-state materials enable lightweight, long cycle life, high-energy density, chemical stable, and high-temperature batteries [1,2]. However, there are several drawbacks like low power density and high ionic resistance. To compensate the low Li-ion diffusion the use of ultra-thin electrodes to achieve high rate capability is required. In general, thin-film batteries enable excellent energy densities, a longer lifetime, a certain degree of flexibility, and extreme lightness [2].

Currently, there is a strong demand for flexible devices which are still operational when bent, folded, compressed, or stretched. Flexible batteries will enable a new generation of wearables and Internet of Everything (IoE) electronic systems. Further applications are smart cards, implantable medical devices, roll-up displays, smart packaging, active radio-frequency identification tags (RFID), smart electronics, and wearable sensors [3,4]. The flexible battery market is forecast to increase dramatically from $69*.*6 million in 2015 to $958*.*4 million by 2022 [5]. The thin-film battery type is expected to dominate the global market. BrightVolt introduced a Li-polymer battery with 14 mAh capacity and ISO 7810 classification. NanoEnergy is a flexible battery consisting of lithium cobalt oxide (LiCoO_2_), lithium phosphorous oxynitride (LiPON), and metallic Li and exhibiting capacities from 0*.*1 to 5 mAh. Although several flexible Li-based batteries are already commercialized, many issues remain and are currently being investigated.

Conventional thin-film batteries are based on brittle or intrinsically inflexible materials. Hence, these batteries are unable to maintain stable power and energy supply and cycling stability for applications under frequent mechanical stress generated by bending, folding, or twisting [3]. Several effects may appear, such as insufficient flexibility and strength of the electrodes, leakage of the liquid electrolyte, poor adhesion between substrate and active materials, and degradation of the mechanical and electrochemical properties at different deformed states [6]. Another issue is the development of lightweight, thin, flexible, and stable encapsulation materials to protect the battery from external influences [3]. In general, freestanding electrodes like carbon nanotube paper, graphene paper, conductive paper, conductive textiles, or inherently non-flexible electrode materials with reduced thickness on a flexible substrate can be applied [3,4,6]. In addition, some electrode materials require annealing treatments at high temperatures which typical flexible substrate materials cannot withstand. As for solid-state electrolytes polymer gels and plastic crystals, among other materials, are under investigation [7].

Here, we report on the development of a flexible thin-film solid-state rechargeable Li battery as a model system to understand the bending impact on the performance of such devices. Ultra-thin layers are deposited on a flexible high-temperature resistant non-conductive substrate. Two hundred-nanometer Li_4_Ti_5_O_12_ (LTO) as cathode, 500-nm LiPON as glassy solid-state electrolyte, and 1-μm Li metal as anode are applied. A detailed systematic study on the battery performance upon bending state is evaluated to gain a better understanding of the resulting effects. Three different bending radii in convex and concave states are tested. The total thickness of the cell including substrate and thin-film encapsulation is below 45 μm. For long-term mechanical protection additional macro-scale encapsulation is tested. Different thin-film encapsulation approaches are considered and battery performance is evaluated.

## 2. Experimental section

A flexible all-solid-state thin-film Li battery consisting of LTO/LiPON/Li was fabricated using sputtering and thermal evaporation techniques. A 40-μm-thick 3 mol% yttria-stabilized zirconia (*ThinE*-*Strate*) ceramic sheet was purchased from ENrG Inc. and used as a flexible substrate. This flexible material withstands processing temperatures up to 1200 °C and shows good insulation properties with a resistivity > 10^14^ Ωm. The low surface roughness of around 20 nm enables thin-film deposition. A metal current collector consisting of 20-nm Ti adhesion layer and 70-nm Pt was deposited by means of e-beam evaporation (*Pfeiffer e*-*gun PLS 580*) applying a shadow mask which separated the anode and cathode contact leads. A 200-nm LTO thin-film electrode of 7.25 cm^2^ area was deposited by RF-sputtering from a 4′′ target (Neyco, 99.5% purity). The deposition was carried out under argon (Ar) flow of 25 sccm. The chamber base pressure was kept at ~3 × 10^−6^ Torr. During deposition the pressure was kept at ~3 × 10^−3^ Torr. The layer was then heat treated for 20 min at 800 °C in oxygen atmosphere using a heating rate of 10 °C/min (*Nabertherm RHTH 120*). The LTO thin film is initially amorphous, and annealing helps to make it crystalline and electrochemical active. Subsequently, a 500-nm LiPON solid electrolyte was deposited by means of reactive RF-sputtering under N_2_ flow of 30 sccm from a 4′′ Li_3_PO_4_ target (Praxair 99.9% purity). The thickness of the layers was monitored by scanning electron microscopy (SEM). Completing the full battery stack a 1-μm Li-metal anode was thermally evaporated in a home-built system. The Li-metal makes contact with its corresponding current collector lead through a shadow mask. During Li deposition the pressure was kept at 10^−5^ Torr. The thickness of the film was monitored by a quartz crystal microbalance (SQM 160, Inficon). Between the various process steps the samples were stored in an Ar-filled glovebox (O_2_, H_2_O < 1 ppm) to avoid undesired reactions with air. Furthermore, the time between process steps was kept to a minimum to obtain preferably clean interfaces. The positive and negative leads were contacted with aluminum tape strips which enable bending test to be performed.

The C-rate performance of the flexible thin-film battery was evaluated by galvanostatic charge and discharge cycles using different current densities from 12 μA cm^−2^ (1 C) up to 120 μA cm^−2^ (10 C) between 0.8 and 2.5 V versus Li^+^/Li. Cyclic voltammograms are recorded at 10 mV s^−1^ before and after the charge–discharge cycles. Measurements were done inside an Ar glovebox using an Autolab potentiostat and Nova software was employed for data collection. This measurement procedure was performed at several bending states of the full battery stack. A home-built bending apparatus was used. The flexible thin-film battery sat on two supports, from which one was fixed and the other was slidable. By moving the supports closer together, the battery was bent in a controlled way. The distance between the ends of the sample (*l*) was measured and the bending radius (*R*_*c*_) was calculated. The anode and cathode electrodes were contacted through Al tape with clamps (see Figs. 2(b,c)).

Different encapsulation schemes were applied to enclose the flexible battery for operation in ambient atmosphere. Atomic layer deposition (ALD) was used to grow a 200-nm Al_2_O_3_ protective layer. Trimethylaluminum (TMA) and H_2_O were used as sources and the deposition was executed at 90 °C to avoid any significant degradation of the battery layers. The flexible battery remained in inert environment since the ALD equipment (Picosun R200) is coupled to an Ar glovebox. In addition, a flexible glass sheet recently introduced by SCHOTT AG (AF 32 eco) was used as additional protection. The ultra-thin glass of 25-μm thickness enables long-term packaging of flexible batteries due to its excellent barrier and mechanical protection properties. Finally, a polydimethylsioxane (PDMS) coating serves as the main encapsulation body of the full flexible battery. The PDMS was prepared in a 10:1 mixing ratio (base to cross-linker) and cured at 90 °C inside an Ar glovebox.

## 3. Results and discussion

### 3.1. Bending impact on battery performance

**Figure 1.**
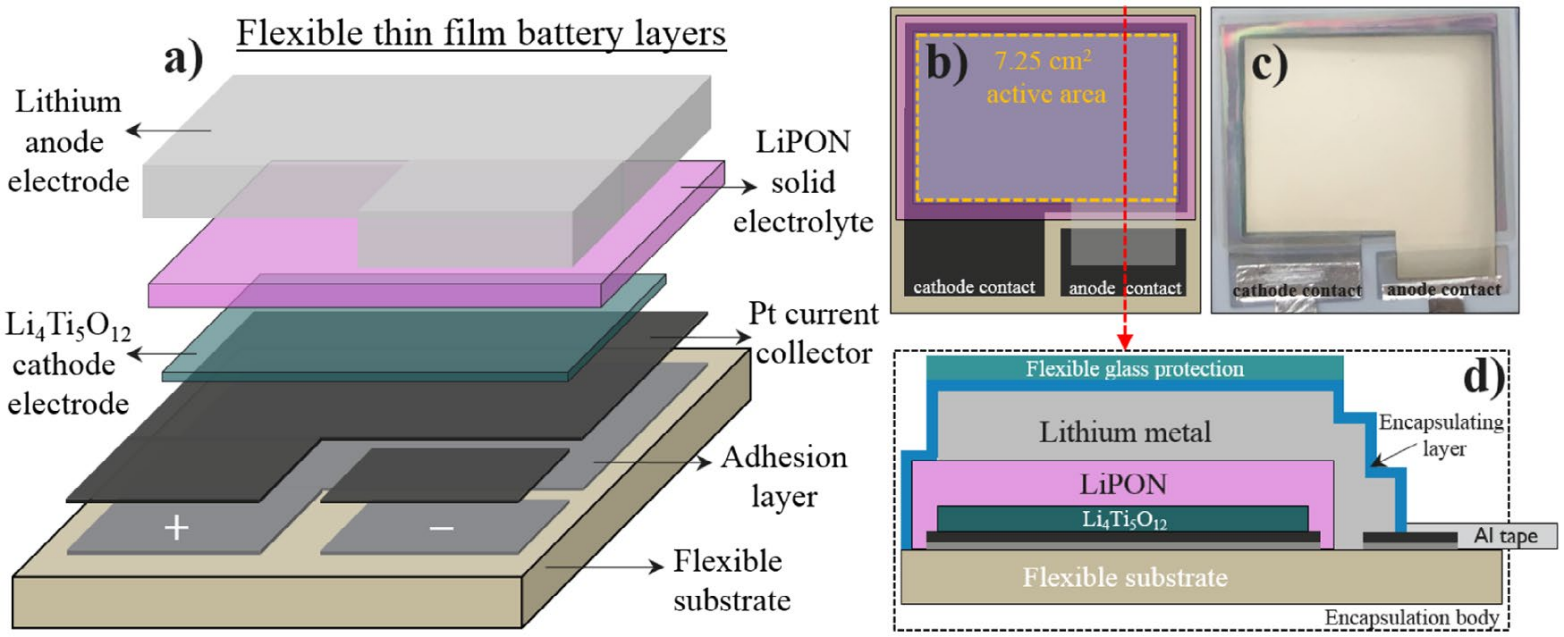
(a) Schematic representation of the flexible thin-film battery layers. (b) Top view representation of the layers showing the 7.25 cm^2^ active area (c) Top view photograph of a real flexible thin-film battery device. (d) Cross section representation of the fully encapsulated battery stack showing all final components.

An operational thin-film battery stack is developed as a model system on a flexible ceramic substrate using physical vapor deposition (PVD) techniques. The total thickness of the metal current collectors and active materials in the battery stack is below 2 μm. The flexible ceramic substrate thickness is 40 μm. To fabricate the full battery each layer is subsequently deposited with specifically designed masks. The processing steps are illustrated in Figure 1(a). This flexible structure with a full thickness below 42 μm is used as a model system to evaluate the C-rate capability upon bending state and the capacity retention of the battery. An electrode active area of 7.25 cm^2^ is defined by the masks used. A top view schematic and picture are shown in Figure 1((b), (c)). The LTO electrode thickness of 200 nm and the active area of 7.25 cm^2^ are used to calculate the volumetric capacity of the battery. This is one of the highest active areas for flexible thin-film Li batteries reported so far and will strongly show the possible effects due to bending. The maximum theoretical capacity of LTO considered here is 600 mAh cm^−3^.

A systematic study on battery performance upon bending state was defined for three different bending states. The degree of bending is referred to the value of the bending radius (*R*_*c*_) of the battery. The curvature radius *R*_*c*_ can be calculated from the geometry by the following:

(1) $$l_{0}=2R_{c}\alpha$$

(2) $$\text{sin}\alpha =\frac{l}{2R_{c}}$$

where *l*_*0*_ is the arc length and α is the arc angle in radians. The resulting equation can be solved numerically to obtain *R*_*c*_:

(3) $$\text{sin}\left(\frac{l_{o}}{2R_{c}}\right)=\frac{l}{2R_{c}}$$

**Figure 2.**
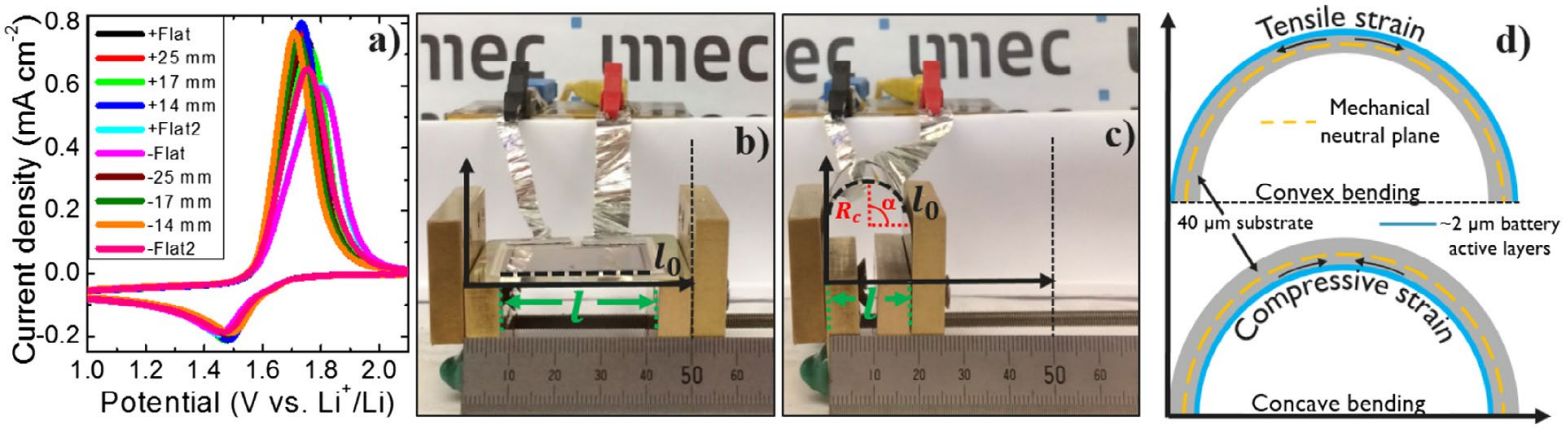
(a) Cyclic voltammograms at a scanning rate of 10 mV s^−1^ for a thin-film all-solid-state flexible battery stack based on Li_4_Ti_5_O_12_/LiPON/Li at different bending states. Photographs of the flexible battery in the bending characterization apparatus on (b) a flat state (*R*_*c*_ = ∞) and c) with curvature radius *R*_*c*_ = 8.5 mm. (d) Schematic representation of the thin-film stack in convex and compressive bending state showing the mechanically neutral plane for both cases and the tensile and compressive strains.

The curvature C of the bent sample is the reciprocal of the curvature radius (*C* = 1/*R*_*c*_). During bending tests the battery is connected by aluminum tape to enable electrochemical measurements in the various bent states. *R*_*c*_ is calculated to the active area of the electrode layers of the battery. For the required parameters, the initial length *l*_0_ (36 mm) of the current collector and the horizontal distance *l* between the ends of the film during bending are measured. This is required since the sample will not bend in a perfect circular arc and gives a more accurate description of the battery layers curvature than considering the total substrate length of 50 mm (see Figure 2((b), (c))). Most reports define the curvature radius by only taking into account the substrate independent of active layer size. Hence, the relation between *R*_*c*_ and performance might be misinterpreted [8–10]. The calculated values of *R*_*c*_ are 25, 17, and 14 mm for a horizontal distance of the active materials (*l*) of 33, 29.5, and 27 mm, respectively. Electrochemical measurements are performed to evaluate the initial capacity retention of the battery and C-rate performance in the flat position and in all bending states. Bending in both convex (+) and concave (−) ways is enabled by flipping the battery. Figure 2(a) shows the initial cyclic voltammograms between 0.8 and 2.5 V versus Li^+^/Li recorded at 10 mV s^−1^ for all bending positions. The sequence of the measurements is shown in the inset. Before and after the various bending radii in a convex state the sample is cycled in a flat condition. Then, the sample is flipped for concave measurements and the same sequence is used. With this procedure, we discard any significant degradation of the battery during cycling. It is noticeable there is a slight shift on the (dis)-charging peaks between bending states showing a ∆V of 90.6 and 43.8 mV for the delithiation and lithiation peaks, respectively. This is an indication of a change in resistance in the battery upon measuring conditions. This is analyzed later in the manuscript. Figure 2((b), (c)) show the flexible battery in the slidable support in a flat state (*R*_*c*_ = ∞) and in a bending state with *R*_*c*_ = 8.5 mm, respectively. Here, we show that the performance of the flexible battery can change depending on its bending state and direction. In general, the impact of bending on thin-film battery performance is not well understood. This battery deformation can influence contact resistance, ion diffusion and structural stability. External forces such as elastic bending of electrode materials influence ion diffusion caused by an inhomogeneous distribution of atoms [11]. Recently, first models for Li-ion diffusion coefficient dependency on mechanical deformation were introduced for LiCoO_2_ electrodes. Chang and Chen have claimed to establish a comprehensive physical model for all-solid-state Li batteries when they undergo bending [12]. To study the relationship between bending stress and battery performance they match their electrochemical model to experimental data for different C-rates obtained by Koo et al. [13]. Although the model provides a link between Li-ion diffusion on the deformation angle it is in contrast with the findings reported here. Since Koo et al. performed battery bending tests by specially locating the active layers at the mechanically neutral plane of the structure where the bending deformation correspond to a zero-uniaxial stress and strain, the bending stress analysis by Chang and Chen might inaccurately describe its effects on thin-film battery performance where active layers are located at a certain distance from the neutral plane. In addition, Ning et al. calculated that the Li-ion diffusion coefficient in LiCoO_2_ can be tuned by lattice strain [14]. It was shown by first principle calculations that tensile strain along the c-axis and *ab*-plane of a LiCoO_2_-layered structure increases the Li-ion diffusion coefficient, while compressive strain leads to inferior diffusion dynamics caused by a larger Li-ion migration energy barrier. Furthermore, the change in contact resistance on bending has been also studied. Gaikwad et al. have shown a contact resistance increase and decrease under compression and tension stresses, respectively [15]. Hence, it can be stated that battery performance dependency on bending direction might be due to strain-type-related change in Li-ion diffusion in the electrode lattice although the physics behind the effect of Li-ion migration by external forces is rather complicated and can be dependent on several factors. Figure 2(d) shows a schematic of the two bending states (convex and concave) used here that cause different strains on the 2-μm-thick active battery layers.

**Figure 3.**
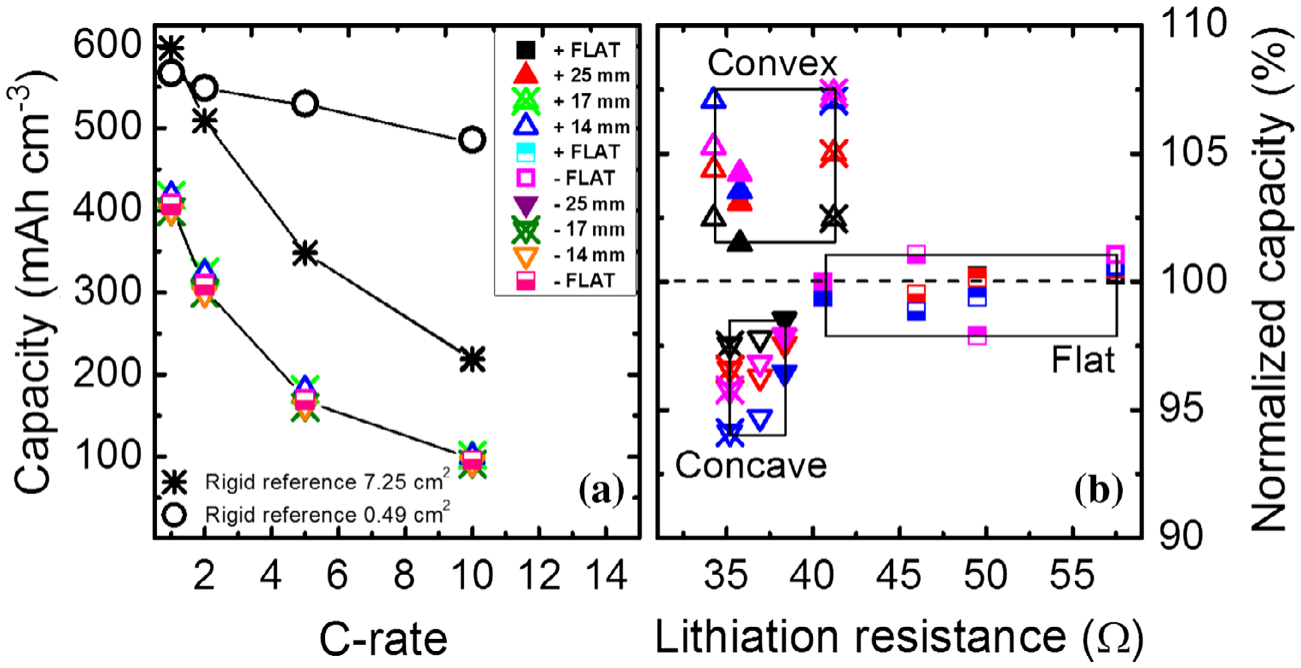
(a) Volumetric capacity as a function of C-rate for the different bending states. Capacities of rigid batteries with various active areas are plotted as reference. (b) Normalized capacity as a function of lithiation resistance for the different bending states at the different C-rates. Squares, up-triangles, and down-triangles represent the flat state, convex and concave states, respectively. Filled, cross-centered and open triangles represent bending states with an *R*_*c*_ value of 25, 17, and 14 mm, respectively. Squares represent the flat states measured before, in between, and after bending.

The total capacity of the flexible battery increases as the tensile strain during convex bending increases, whereas compressive strain in concave bending shows a capacity decrease. Figure 3 shows (a) the volumetric capacity referred to the LTO obtained as a function of C-rate for the different bending states of the battery and (b) the normalized capacity retention as a function of lithiation resistance for the different bending states at various C-rates applied. To quantify the C-rate performance the battery is cycled by applying different current densities. The C-rates applied here are 1 C (87 μA), 2 C (174 μA), 5 C (435 μA), and 10 C (870 μA). The detailed (de)-lithiation curves for the various bending states are shown in the supporting information (Fig. S1). First of all, it can be seen that the flexible battery can reach 70% of the maximum theoretical capacity for LTO (600 mAh cm^−3^) at 1 C. In detail, the capacities are in the range of 418 mAh cm^−3^ (1 C), 310 mAh cm^−3^ (2 C), 170 mAh cm^−3^ (5 C), and 95 mAh cm^−3^ (10 C). For comparison, a rigid reference is fabricated using the same mask design, active area, and materials, but deposited on a Si substrate with 500-nm SiO_x_. The rigid reference achieves a total capacity matching the maximum theoretical capacity for LTO at 1 C (Fig. 3(a)) and follows roughly the same decay rate at higher C-rates as the flexible version. This comparison shows that there is an intrinsic capacity loss for the flexible battery. Surface roughness measurements for both rigid and flexible samples were performed to investigate the difference in capacity. Figure S2 shows that the current collector surface of the flexible sample has a root mean square (rms)value of 22 nm, which implies that it basically follows the roughness of the ceramic substrate (*ENrG Thin E*-*strate*) [16]. In contrast, the current collector of the rigid reference sample is very smooth (rms = 0*.*44 nm). The large difference in surface roughness will continue in the subsequent layers, which would affect the electrochemical performance of the thin-film battery. The higher interface roughness of the flexible battery can be clearly seen from a cross-sectional SEM image shown in Fig. S3. The lower capacity obtained at 1 C for the flexible battery may be explained by the rough surface of the substrate, which can lead to a higher resistance in-between and within the subsequent layers. First attempts to improve the surface roughness of the flexible ceramic by depositing a buffer layer onto the substrate lead to a slight improvement regarding the roughness. However, a sufficient method to match the surface quality of the rigid reference needs to be investigated in future work. To show the electrochemical performance of the battery excluding surface roughness and geometrical effects, a smaller rigid reference with an active area of just 0.49 cm^2^ was fabricated. The C-rate dependence of the small area reference is also shown in Figure 3(a). The maximum capacity is close to theoretical values at 1 C, while at 10 C the retained capacity is still around 85%. We assume that the larger electrode area (factor of 15) and the surface roughness of the substrate is the main cause of the inferior C-rate performance of the flexible version. The large applied currents resulting from the increased active area may not efficiently spread over the anode area or enable parasitic current paths.

Figure 3(a) also shows a slight variation of the capacities obtained for the flexible thin-film battery at each bending state. To study this subtle variation more detailed the capacities are normalized to the total capacity obtained in the non-bent state. In Figure 3(b), it can be clearly seen that the convex bent battery can achieve higher capacities, while the concave bent battery shows lowest capacities when referenced to the flat state capacities obtained at the beginning, the middle, and end of the measurement cycle. Due to this measurement order, degradation during cycling causing this effect can be excluded. All measurements reveal excellent efficiencies above 99*.*3%, which enables a high-cycle life.

An increase and decrease in capacity with convex and concave bendings, respectively, is clearly visible for all C-rates. Figure 3(b) shows the normalized capacity as a function of the lithiation resistance at the various bending states and current densities applied. The (de)-lithiation resistances are extracted by plotting the plateau values of the cell voltage during charge/discharge (Fig. S1) for the corresponding currents applied at the various C-rates. The slope of a linear fit of the resulting V–I graph gives the resistance value. The capacity is normalized to the capacity obtained at 1 C for the first measurement in the flat state. Table 1 shows the capacity variations for all the bending states at the different C-rates.

**Table 1.** Values of capacity change at convex and concave bending states with bending radii of *R*_*c*_ = 25, 17, and 14 mm at different C-rates (1, 2, 5, and 10 C).

C – rate	Capacity @ convex (%)			Capacity @ concave (%)		
	+25 mm	+17 mm	+14 mm	−25 mm	−17 mm	−14 mm
1 C	+1.49	+2.45	+2.48	−1.50	−2.45	−2.26
2 C	+3.04	+5.05	+4.36	−2.43	−3.45	−3.74
5 C	+3.54	+7.06	+7.03	−3.57	−5.88	−5.36
10 C	+4.23	+7.32	+5.35	−2.12	−4.19	−3.19
Average	+3.07	+5.47	+4.80	−2.40	−3.99	−3.63

Overall, the change in capacity increases with higher C-rates applied, except for a R_c_ of 14 mm. The change in capacity for the different bending states shows that tension strain in the flexible thin-film battery is beneficial for Li-ion diffusion across the active layers. The concave bending state might induce a larger Li-ion diffusion energy barrier which can be seen from the reduction in capacity. One of the possible causes of this capacity change could be related to a change in resistance. Considering the lithiation resistance, it is noticeable that the capacities obtained for the flat states are not affected by the increase in resistance. Regardless the lithiation resistance, which varies between 40.6 and 57.5 Ω, the capacities are in the same range varying within ±1.25%. Furthermore, the flexed states show larger differences in capacity for convex and concave bending up to a maximum of 13%, while the resistance is in the same range (∆R ± 3.5 Ω). Hence, it can be concluded that induced film stress is probably the main cause of capacity variation. Whereas, an increase of tensile stress during convex bending has a positive effect on the capacity, compressive stress decreases the obtained capacities. This trend of bending impact on capacity was the same for a similar sample with an identical layer stack, not shown here. However, the absolute capacity values differ, which is due to initial material quality and variation during material preparation.

These results are in contrast with some findings from other groups. Koo et al. demonstrated a decrease of about 10% in capacity with increasing convex bending [13]. However, the mechanically neutral plane is initially located in the active layers due to the substrate transfer approach applied. This might have a significant impact on the overall results and interpretations of the bending effect. Usually, the single neutral plane is located at the geometric center of a multilayered system. For a flexible thin-film battery, the position of the neutral plane can be tuned by final flexible encapsulation but may differ with the design of the device. In addition, the distance of each layer to the geometrical center of the device will vary making further layers more susceptible to bending effects. Also, a single mechanical neutral plane will only protect a single layer at a time. Multiple neutral planes designs have been proposed for several flexible electronic applications [17]. The model system proposed here consists of a neutral plane located outside the active battery layers where a bending impact is to be expected.

**Figure 4.**
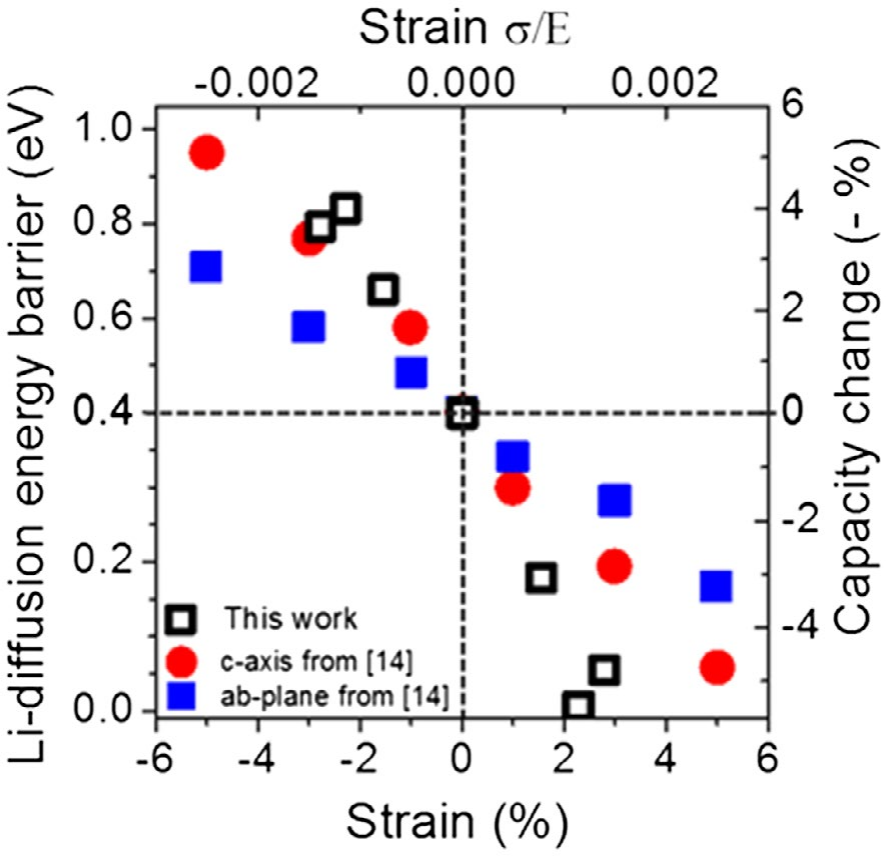
Relationship between the Li-diffusion energy barrier as a function of strain applied according to the simulation from [14] (red circles and blue squares). The open black circles represent the average change in capacity obtained from our flexible thin-film battery bending experiments as a function of strain (stress).

The data by Koo and coworkers were found to be in agreement with a model of Li-ion diffusion proposed by Chang and Chen [12]. Interestingly, this seems to be the case as well for strain induced effects studies on the spinel structure of LiMn_2_O_4_. Yan et al. identified that the energy barrier for Li-ion migration in λ-MnO_2_ can be decreased significantly by compressive strain, thus enhancing the Li-ion diffusion coefficient as well as the electrical conductivity [18]. One explanation to this was the change in stability of the Jahn-Teller type polaron due to the strain causing a significant volume expansion. The differences in volume expansion may be linked as well to the electrochemical performance upon bending state. In contrast, Li_4_Ti_5_O_12_ is a well-known zero-strain insertion material with a spinel structure. In addition, a lattice mismatch between an electrode material and the underlying substrate will induce a strain. This is known to change the electronic structure of transition metal oxides [19]. Thus, a clear analysis of bending stress as a function of distance to the mechanical neutral plane, electrode volume expansion, and substrate lattice needs to be addressed in order to proper understand its effect on the components of a thin-film battery as well as a dependency on electrode thickness. Figure 2(d) illustrates that the mechanical neutral plane for the measurements presented here relies totally on the substrate. This can give contrasting results between bending test and electrochemical performance when compared to previous studies on different flexible batteries and materials where the neutral plane is differently located. Pereira et al. investigated reasons for decrease in capacity for bending radii below 181 mm [20]. In their work cracks in the microstructure of the thin films, disaggregation of the *LiPON* electrolyte, and breakage of the mica substrate caused the decreasing capacity. On the other hand, an improvement in capacity of approximately 10% and a reduced charge-transfer resistance was observed by Gaikwad et al. for tensile strain [15]. An increased capacity was also shown by Kammoun et al. [21]. A similar result was obtained by Li and Ardebili [10]. This effect is different for thin-film solid-state batteries. Glenneberg et al. demonstrated an increased capacity for a bending radius of 20 mm for a thin-film battery on a flexible polymer substrate [22]. Slightly better capacity retention for a flexed state with 5-mm bending radius was observed by Kutbee et al. [7]. The change in capacity as a function of bending state might be also material dependent. Here, we try to relate the experimental observations on capacity change as a function of bending state to the variation of energy barriers for Li-ion diffusion as a function of applied external strain obtained for LiCoO_2_ electrode by Ning et al. using density functional theory (DFT) [14]. The effect of strain (or stress) to the Li-ion dynamics shows that the migration energy barrier for Li-ions increases upon compressive strain, while it decreases with tensile strain. The decrease of the energy barrier will enhance Li-ion diffusion, thus observing an enhancement in rate performance and capacity. This view is in agreement with our experimental findings. In addition, our results demonstrate the coupling between electrochemical kinetics and mechanical generated stress following the chemomechanical modeling of lithiation processes. It is known that lithiation kinetics by itself modulates stress in electrode materials with large volumetric expansion which can lead to a stress-mediated lithiation retardation [23,24]. External forces are also known to modulate lithiation kinetics by creating chemical potential differences between electrodes due to induced stress [25]. The effect shown here demonstrates the modulation of Li-ion migration between tensile and compressive states. While tensile stress favors Li-ion migration, large induced compressive stress shows lithiation retardation where active materials become inaccessible to Li-ions. Figure 4 shows the simulation data taken from [14] and compares it to our capacity versus strain results. The stress generated due to the bending state in our measurements can be calculated by assuming the simple bending theory by the following equation:

(4) $$\sigma =\frac{\mathrm{Ed}}{R_{c}}$$

where *E* is the Young modulus (N/m^2^), *d* is the distance of the electrode surface from the neutral plane (*m*), and *R*_*c*_ the bending radius defined previously in equation 3. Taking the full battery stack configuration (see Figure 1) and the thickness of each layer, the mechanically neutral plane is situated at 20.895 μm, hence the distance of the LTO electrode surface to the neutral plane is *d* = 19.295 μm. According to equation 4, the resulting stress values for each bending radius are σ = 155, 228, and 277 MPa for *R*_*c*_ = 25, 17, and 14 mm, respectively. One can derive the force applied for each bending state considering the relation σ *= F/A,* where *F* is the force applied and *A* the cross-sectional area (36 mm × 41.79 μm) and the strain to stress relation (ε = σ/E). The maximum strain limit considered here is 0.003 according to the specifications provided by the supplier for failure breaking tests [16]. This is calculated by assuming a Young’s modulus (*E*) of 200 GPa which is defined for the flexible ceramic substrate and by considering a failure probability of 98% obtained when applying a strength of 1.2 GPa. In addition, the elastic properties of some electrode materials including LTO have been analyzed using DFT calculations by Qi et al. [26]. The Young’s modulus obtained for fully delithiated and lithiated LTO was found to be *E* = 181 and 209 GPa, respectively. Although the value of *E* can change with Li concentration we consider that the value used is a good approximation for the defined calculation. Lastly, the maximum strain limit obtained is linked to the 5% maximum lattice strain from Ning et al. [14]. It is assumed that the maximum capacity change is 5.47% (Table 1) for both conditions of bending. That is, a maximum change of 5.47% is linked to the higher energy barriers for Li diffusion at 1 eV and to a free Li diffusion pathway (Energy barrier = 0). In addition, it is assumed that under zero strain the change in capacity is 0. Details of the calculation procedure for the introduced parameters are displayed in the supporting information.

The observable change in capacity upon bending state seems to be strongly related to the change in Li-ion diffusion in the thin-film electrode lattice caused by the positive and negative stresses applied, this is according to our first approximations and following the above assumptions. Although there are several other factors that play an important role in the battery performance (i.e. roughness, active area, geometry, substrate, stress symmetry, Li concentration, and neutral plane location) the bending applies an external force to the crystal structure of the electrode material and seems to be the main cause of the change in battery performance reported here. Since this effect is related to the Li-ion diffusion through the crystal structure this will make the effect material-dependent. Electrode materials with large volume change during cycling, such as Si, are known to be susceptible to chemomechanical degradation. Also, the behavior will be affected by the layers above the electrode material, their thickness, and how the Li-ion diffusivity travels in those layers and interfaces. It is clear that understanding bending effects on battery performance will play an important role when designing and fabricating new energy storage devices. Our model system shows that, to a first approximation, the change in capacity in flexible batteries might be sufficiently related to Li-ion diffusivity upon stress.

### 3.2. Thin-film battery encapsulation

A full flexible encapsulated thin-film battery was successfully achieved reaching up to > 500 cycles of operation before breakdown while in air. All previous experiments were performed in an Ar-filled glovebox since especially the Li-metal anode is highly sensitive to air. Hence, the battery needs to be encapsulated. The high reactivity of Li-metal and the comparable low melting point of 180 °C restrict the choice of encapsulating materials and deposition methods. Here, we make use of ALD to deposit a 200-nm protective layer of Al_2_O_3_ on the flexible battery. Successful ALD surface treatments on Li-metal at 100 °C have been already shown [27]. Kozen et al. demonstrated suitable protection of Li foil surface for 20 h with a 36-nm alumina layer grown with O_2_ plasma and a deposition temperature of 150 °C [28]. Here, a low-temperature process is required due to the low melting point of Li and to maintain the glassy character of the LiPON solid electrolyte. An ALD deposition process of Al_2_O_3_ is done at 90 °C using TMA and H_2_O as material sources.

**Figure 5.**
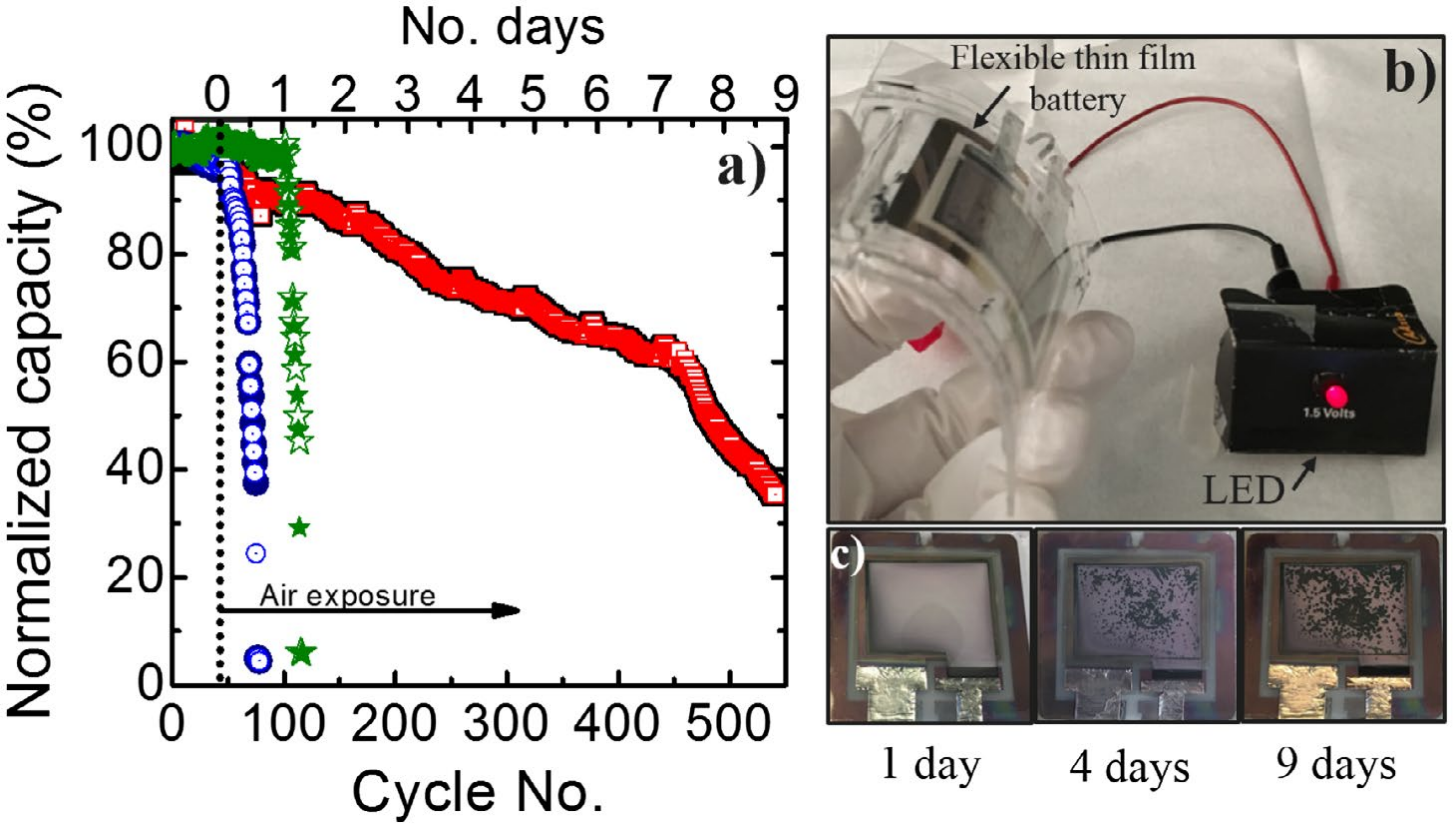
(a) Flexible thin-film battery cycling before and after air exposure. Blue circles, green stars and red squares represent the cycling of a Li_4_Ti_5_O_12_-based flexible battery with (1) Al_2_O_3_ coating, (2) Al_2_O_3_ + PDMS, and (3) Al_2_O_3_ + thin glass sheet + PDMS encapsulation, respectively. (b) Photograph of the flexible thin-film battery at day 4 of air exposure working as a power source for an LED. (c) Top view photographs of a fully encapsulated battery (Al_2_O_3_ + thin glass + PDMS) showing the morphology changes of the Li-metal anode at 1, 4, and 9 days of air exposure.

Although the final device allows bending, the encapsulated flexible thin-film batteries are cycled in a flat state. Figure 5(a) shows the cycling of flexible thin-film batteries with different encapsulation schemes. A comparison is made using the flexible thin-film battery structure including (a) only Al_2_O_3_ protective layer, (b) Al_2_O_3_ protective layer and PDMS encapsulation, and (c) Al_2_O_3_ protective layer, flexible thin glass sheet, and PDMS encapsulation. The immediate failure of the battery for a sample without any protection and direct exposure of the Li electrode to air is not shown here. The measurements before and after capping show that the efficiency is not affected by the 200-nm alumina protective layer since it remains above 99%. There is a small capacity decrease before and after alumina deposition of 1.7% which indicates that the 90 °C process has no significant negative effect on the Li-metal anode and the amorphous LiPON electrolyte since it is within the measurement variations.

When using only an Al_2_O_3_ protective layer, cycling in air reveals a drop of 10% for the first 10 cycles. After approximately 4 h of operation a strong decrease occurs. The capacity starts to drop dramatically after 8 h until it reaches almost zero before 50 cycles at 1C. This is represented with the blue circles in Figure 5(a). The battery remains stable at the beginning of the measurements when it is cycled in an Ar-filled glovebox. The results show that the alumina layer is not sufficient for proper protection of the battery when operating in air, in contrast with some findings from Kozen et al. on coatings on Li-metal foils [28]. The effect of Al_2_O_3_ layers on Li-metal anodes has been studied also by Kazyak et al. who observed an improved stability when using proper preparation conditions [27]. Nevertheless, according to their findings specific combinations between thermal treatments and chemical reactions can delay or accelerate battery failure. We assume that there is an increase of inactive Li material after alumina deposition related to the creation of LiAlO_x_ and LiO_x_ layers, which are thermodynamically stable and are poor Li-ion conductors. The Li-metal anode roughness, the measurement conditions, and the ALD deposition sources might play an important role on the effectiveness of the capping layer. Furthermore, a ~1-mm thick PDMS encapsulation body is added to an Al_2_O_3_-coated flexible battery to observe if there is any improvement on the cyclability (green stars in Figure 5(a)). Also, the battery remains stable before air exposure. After exposure it continues to be stable. There is a 2.5% loss of capacity after the first 40 cycles until an abrupt drop occurs at 115 cycles indicating the battery failure. These results demonstrate that adding the PDMS enclosure does not significantly improve the cycling stability. Although, it is known that PDMS does not block moisture, here it was intended to serve as a layer delaying battery failure. However, there was no noteworthy improvement. As previously mentioned, the addition of encapsulating layers changes the mechanically neutral plane and can therefore also change the electrochemical performance of the battery. The dependence of battery performance on changing the neutral plane is not the scope of this study, but the stability on encapsulation approach. When analyzing the last flexible battery encapsulation, which includes a novel flexible thin glass sheet (25 μm), a significant improvement is observed. The cycling behavior of the fully encapsulated flexible thin-film battery (Al_2_O_3_ + glass sheet + PDMS) is shown by the red squares in Figure 5(a). A cross-section schematic of the full battery device is shown in Figure 1(d). The battery is cycled in a flat state and can remain operational still reaching 60% of its initial capacity for more than 450 cycles in air with a coulombic efficiency above 99%. Hence, it can be stated that the additional encapsulation sheet improves the time period dramatically up to at least a factor of 4. Furthermore, the performance of the introduced flexible encapsulation is in the same range as rigid epoxy-based encapsulation [29].

**Figure 6.**
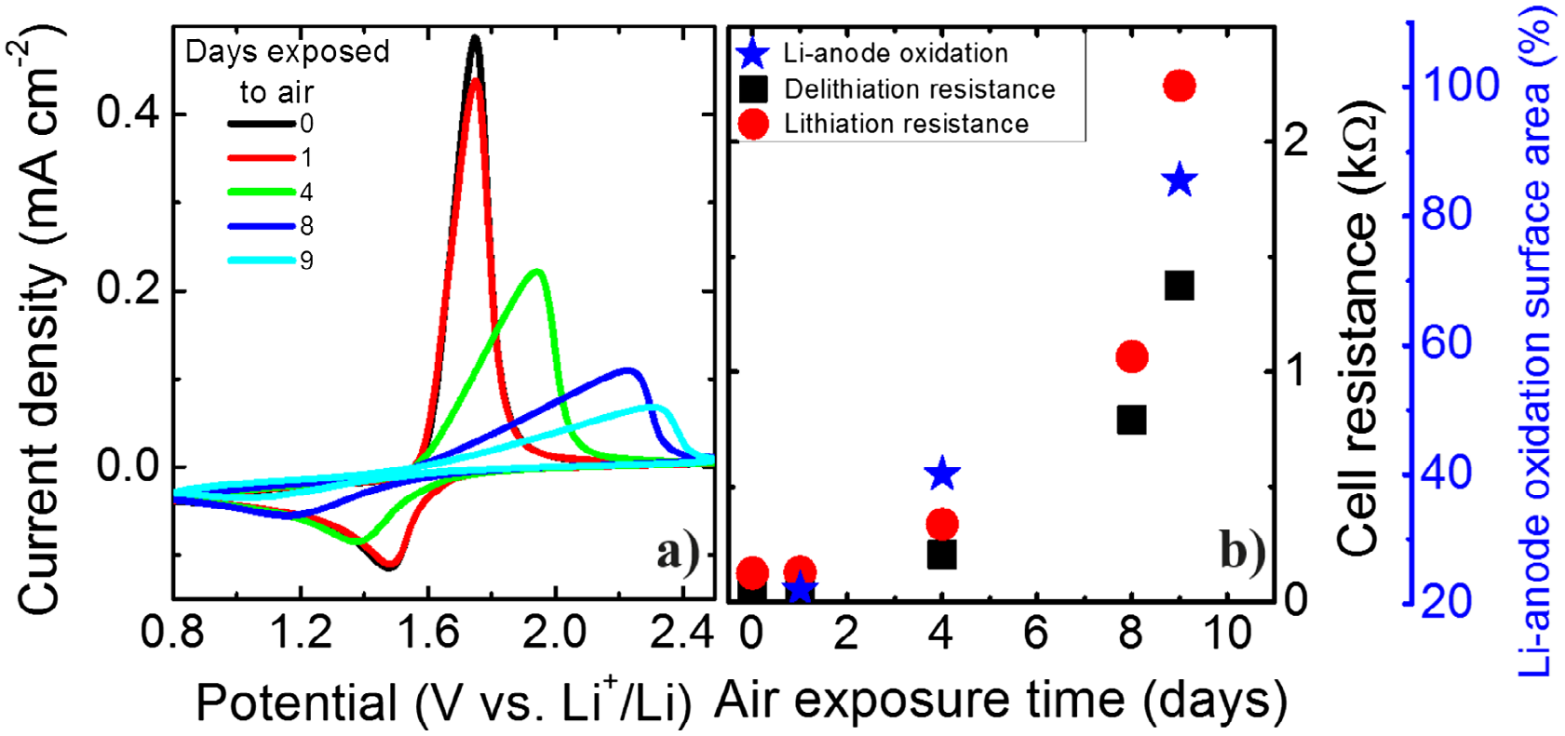
(a) Cyclic voltammograms at a scanning rate of 10 mV s^−1^ for a thin-film all-solid-state flexible battery stack based on Li_4_Ti_5_O_12_/LiPON/Li at different exposure days to air. (b) Cell resistance and Li-metal anode oxidized surface area as a function of air exposure.

To better characterize the effect of exposure to air of the fully encapsulated flexible battery the CVs measured at different times are compared. Figure 6(a) shows that after one day of exposure to air almost no change in the peak shape is visible, although the delithiation peak is slightly smaller. However, after four days the peaks shift, decrease, and broaden, which indicates increasing overcharging due to higher internal resistances. This trend continues for the remaining days of air exposure. For this reason we compare the (de)-lithiation resistances. The resistance is obtained from the slope of the respective peak in the CVs and is shown in Figure 6(b). For all measuring points the lithiation resistances are higher than the delithiation ones and both resistances show an exponential growth with air exposure time. Directly after exposure to air the resistances are around 38 Ω (delithiation) and 125 Ω (lithiation). After 9 days the resistances increase to around 1375 Ω (delithiation) and 2245 Ω (lithiation). This large increase in cell resistance can indicate extensive oxidation of the Li-metal anode due to the moisture/oxygen diffusion through the encapsulating parts and interfacial resistance between electrodes and electrolyte. Also, this long cycling could lead to the development of ‘dead’ inactive Li regions due to plating and stripping of Li-metal. Figure 5(c)–(e) shows a top view of the fully encapsulated flexible battery showing an obvious oxidation evolution on the Li-metal anode. The flexibility and mechanical integrity of the encapsulated thin-film battery are demonstrated in Figure 5(b). Even after 4 days of air exposure the battery is operational and can easily light up a light-emitting diode (LED) even when bent. The Li metal anode surface is analyzed with ImageJ software to calculate the degree of oxidation from the images taken in days 1, 4, and 9 (Figs. 5(c)–(e)). The area of the Li-metal is only taken into account by fixing the threshold from the 32-bit image. The Li-anode oxidized surfaces obtained are 22, 40, and 86%. There is a similar trend when comparing the oxidation of the Li-metal anode to the increase in cell resistance. This can serve as a confirmation that the cell resistance of the thin-film battery mainly arises from the oxidation of the Li-metal anode.

## 4. Conclusions

In conclusion, an operational flexible thin-film solid-state Li battery was demonstrated as a model system to understand the bending behavior in all-solid-state thin-film batteries. The battery consists of a 20 nm/70 nm Ti/Pt current collector, a 200-nm Li_4_Ti_5_O_12_ cathode electrode, a 500-nm LiPON solid electrolyte, and a 1-μm Li-metal anode electrode. A novel high-temperature resistant ceramic sheet is applied as a substrate. The flexible battery achieves 70% of the maximum theoretical capacity for LTO. The surface roughness of the flexible ceramic substrate was identified as the main cause of the decreased capacity compared to a rigid reference on a Si substrate. The flexible thin-film Li battery was bent in a convex and concave way with three different bending curvatures, and the cell resistance and rate performance were analyzed in detail. Functionality is ensured up to an active layer bending radius of at least but not limited to 14 mm. Convex bending (tensile strain) increases the capacity and improves the C-rate performance. The maximum increase obtained in convex mode is 7.32% in comparison to the capacity obtained in a flat state. In contrast, concave bending (compressive strain) lowers the capacity by up to 5.88%. The overall trend is the same for all C-rates applied. Here, we show that our experimental data can be reasonably fitted to a first approximation to a previously reported DFT model monitoring the Li-ion diffusivity energy barrier as a function of applied strain in an electrode material and compare it to the average capacity change upon bending state of the full flexible battery. This effect also supports the view of lithiation retardation due to mechanical stress. While tensile stress favors Li migration, compressive stress slows down Li diffusivity leading to a stress-modulated lithiation retardation. Although, it is clear that more work needs to be done to comprehend the effects that flexible batteries undergo while bent, these results may lead to a better understanding of stress-related phenomena in flexible thin-film batteries.

Several encapsulation schemes were investigated in order to determine the most suitable barrier for the thin-film flexible battery. A layer stack consisting of a 200-nm alumina layer in combination with an advanced flexible thin glass sheet and PDMS protection enables an operational flexible battery for at least 500 cycles in atmospheric conditions while allowing sufficient bending of the device. It was demonstrated that after 4 days of air exposure the bent sample was able to power an LED. The results reported here on the development of an encapsulated flexible thin-film solid-state Li battery will enable further improvements toward all-in-one flexible systems for upcoming consumer electronics.

## Disclosure Statement

No potential conflict of interest was reported by the authors.

## Funding

This work was supported by the Horizon 2020 Framework Programme [grant number 658057].

## Supplemental data

Supplemental data for this article can be accessed https://doi.org/10.1080/14686996.2018.1468199.

## Supplementary Material

suppl.pdf
