# Supplementary material for: Bending impact on the performance of a flexible Li4Ti5O12-based all-solid-state thin-film battery
Source: Sci Technol Adv Mater. 2018 May 25;19(1):454–64. doi: 10.1080/14686996.2018.1468199 (PMC5974753; doi:10.1080/14686996.2018.1468199)
Supplement: suppl.pdf [file TSTA_A_1468199_SM9103.pdf]

Bending impact on the performance of a flexible $Li_4Ti_5O_{12}$-based all-solid-state thin film battery

Alfonso Sepúlveda[a,‡], Jan Speulmanns[a], Philippe M. Vereecken[a,b]

[a]*Imec, Kapeldreef 75, 3001 Leuven, Belgium*

[b]*Centre for Surface Chemistry and Catalysis, University of Leuven, Kasteelpark Arenberg 23, 3001 Leuven, Belgium*

‡Corresponding Author: alfonso.sepulvedamarquez@imec.be

Supporting Information

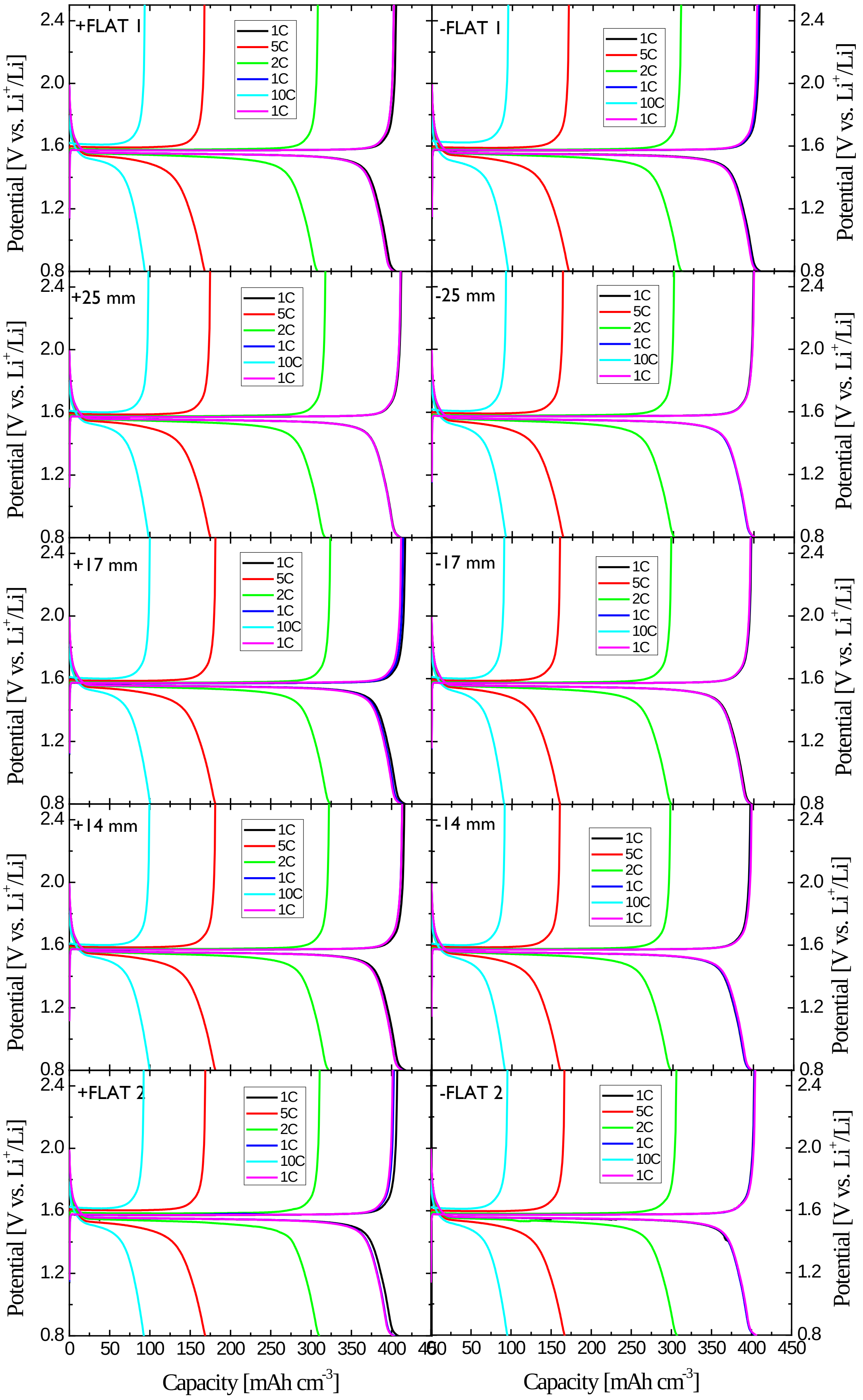

Figure S1: Charge and discharge cycles for the flexible all-solid-state battery at different C-rates for different bending states.

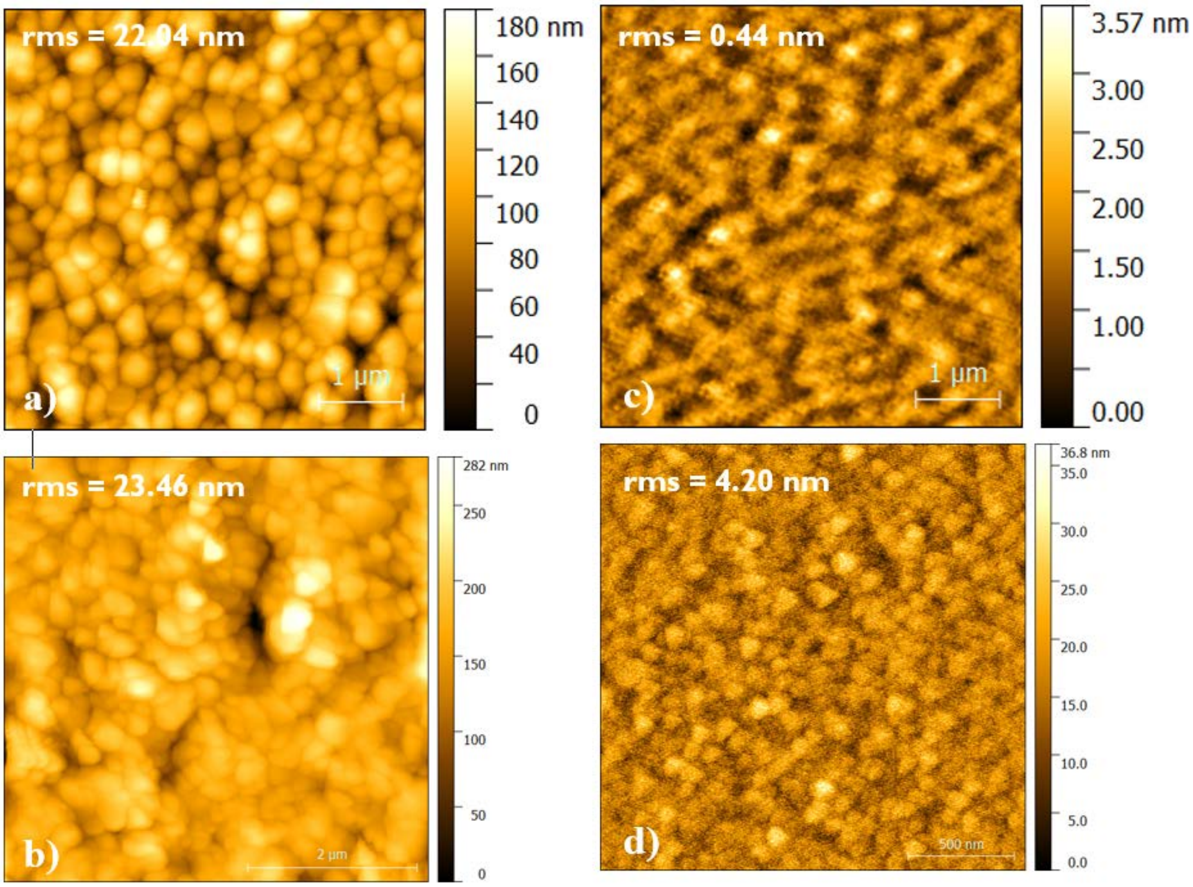

Figure S2: AFM measurements done on a) bare flexible ceramic substrate, b) Flexible ceramic substrate + 200 nm LTO layer, c) bare SiOx substrate and d) SiOx substrate + 200 nm LTO layer.

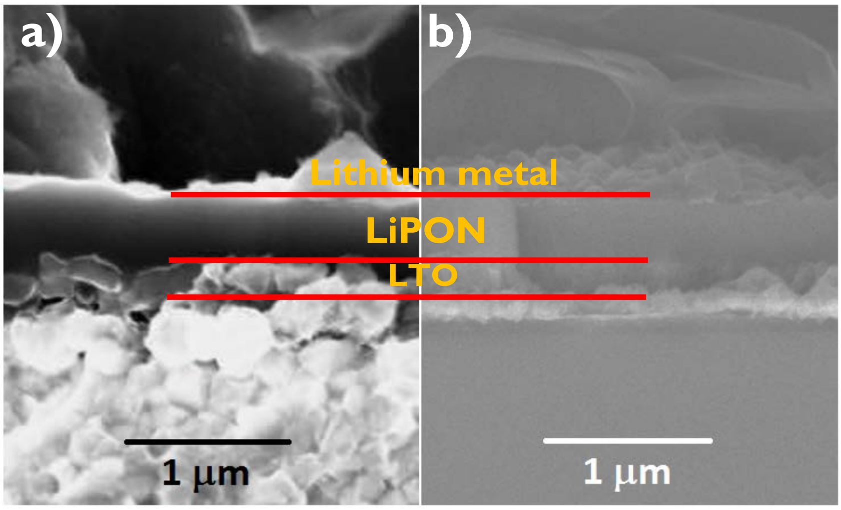

Figure S3: Cross section SEM of full battery stack on a) a flexible ceramic substrate and b) a rigid SiOx/Si substrate.

Calculation procedure to define Figure 4

Layer thicknesses and values:

Flexible substrate: 40 μm
Ti adhesion layer: 20 nm
Pt current collector: 70 nm
$Li_4Ti_5O_{12}$ cathode electrode: 200 nm
LiPON solid electrolyte: 500 nm
Lithium metal anode electrode: 1 μm
Total thickness : 41.79 μm
Mechanical Neutral Plane situated at: 20.895 μm
(d) $Li_4Ti_5O_{12}$ electrode distance to neutral plane: 19.295 μm
(E) Young's Modulus: 200 Gpa
(Rc) Bending radii: 25, 17 and 14 mm

The stress for each bending radius is calculated by:

$$\sigma = \frac{E \cdot d}{Rc}$$

Resulting in:

σ = 154, 227 and 276 MPa for *Rc* = 25, 17 and 14 mm, respectively.

The force applied at each bending state is considered following the relation:

$$F = \sigma A$$

where A is the cross-sectional area of the active materials in the flexible battery

A = 36 mm x 41.79 μm = 1.5044E-6 m^2

Giving as a result for the Force as:

F = 232, 341 and 415 Newtons for *Rc* = 25, 17 and 14 mm, respectively.

The value for strain can be defined with the strain to stress relation:

$$\varepsilon = \sigma/E$$

Resulting in values of strain of:

ε = 0.000772, 0.00114 and 0.00138 for *Rc* = 25, 17 and 14 mm, respectively.

This values are plotted in Figure 4 and are related to the corresponding capacity changes for each bending radius depicted in average values from Table 1. To link it to the results from

Ning et al.[14] the maximum strain limit is calculated according to the specifications provided by the supplier from the Weibull distribution obtained from the physical and mechanical properties from https://www.enrg-inc.com/technology.

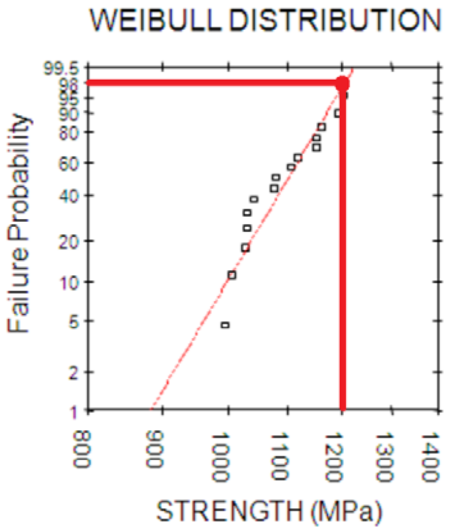

Figure taken from https://www.enrg-inc.com/technology

This is calculated by assuming a Young's modulus (E) of 200 GPa which is defined for the flexible ceramic substrate and by considering a failure probability of 98 % obtained when applying a strength of 1.2 GPa. Using the cross sectional area (A) of 0.8E-6 m^2. The force obtained at 1.2GPa strength by the relation $F = \sigma A$, results in: 96 Newtons. When applying this same force in the geometry of our thin film flexible battery (A = 1.5044E-6 m^2) the resulted stress achieved is $\sigma = 638$ MPa. Next, the value for strain can be defined with the strain to stress relation ($\varepsilon = \sigma/E$) resulting in a maximum strain $\varepsilon = 0.003$. This value is linked to the 5 % maximum lattice strain from Ning et al.[14] It is assumed that the maximum capacity change is 5.47 % (Table 1) for both conditions of bending. That is, a maximum change of ± 5.47 % linked to the Lithium diffusion energy barrier of 1 eV and to a free Lithium diffusion pathway (Energy barrier = 0). In addition, it is assumed that under zero strain the capacity the change in capacity is 0.
